# The *Oral Health in America* Report: A Public Health Research Perspective

**DOI:** 10.5888/pcd19.220067

**Published:** 2022-09-08

**Authors:** Jane A. Weintraub

**Affiliations:** 1 University of North Carolina at Chapel Hill Adams School of Dentistry and Gillings School of Global Public Health, Chapel Hill, North Carolina

## Introduction

In December 2021, the National Institutes of Health, National Institute of Dental and Craniofacial Research, released its landmark 790-page report, *Oral Health in America: Advances and Challenges* (1). This is the first publication of its kind since the agency’s first *Oral Health in America: A Report of the Surgeon General* described the silent epidemic of oral diseases in 2000 (2). This new, in-depth report, an outstanding resource, had more than 400 expert contributors. Its broad scope is exemplified by its 6 sections (Box), each of which includes 4 chapters: 1) Status of Knowledge, Practice, and Perspectives; 2) Advances and Challenges; 3) Promising New Directions; and 4) Summary. In this essay, I provide a public health research perspective for viewing the report, identify some advances and gaps in our knowledge, and raise research questions for future consideration.

Box **Section Titles, *Oral Health in America: Advances and Challenges *(1)**

1. Effect of Oral Health on the Community, Overall Well-Being, and the Economy

2A. Oral Health Across the Lifespan: Children

2B. Oral Health Across the Lifespan: Adolescents

3A. Oral Health Across the Lifespan: Working-Age Adults

3B. Oral Health Across the Lifespan: Older Adults

4. Oral Health Workforce, Education, Practice, and Integration

5. Pain, Mental Illness, Substance Use, and Oral Health

6. Emerging Science and Promising Technologies to Transform Oral Health

## Data Needed

A recurring theme in the report is the need for many types of data, from microdata — the molecular, nanoparticle level — to macrodata — the population and global level. Data are needed to guide public health policies and programs at the federal, state, and local levels. Future research using big data from multiple sources (eg, community health needs assessments, surveillance systems, GIS mapping, electronic health records, practice-based research networks) will provide timely, population-based information to evaluate and drive changes to policy and delivery systems and oral health advocacy efforts.

This new report includes descriptive national data from 3 cycles of the National Health and Nutrition Examination Survey (NHANES). To continue monitoring national oral health surveillance data and trends, oral health data need to be included routinely in NHANES and in other large national studies. Too often, questions about oral health are missing from surveys, or clinical oral health data are not collected. For example, very little about oral health was included as part of the planned data collection protocol for the National Institutes of Health *All of Us *Research Program. This program aims to collect health information from 1 million people (3). Local and state data are often outdated, incomplete, or unavailable. Most oral health data are cross-sectional and are useful for studying trends and associations, but population-based longitudinal data to study causality and the effectiveness of interventions and policies are sparse.

How does oral health care improve other health conditions? Proprietary claims data from insurance companies (4) show the inter-relationship between treatment of periodontal disease and systemic conditions, but secondary data analysis has many limitations and confounding factors. Clinical trials show that periodontal treatment improves glycemic control among people with diabetes (5), but long-term outcome assessments are lacking. We need more answers to convince policy makers and payers about the importance of including comprehensive adult oral health services in publicly financed programs such as Medicaid, which is currently lacking in many states, and Medicare, where those services are missing altogether.

## Health Disparities and Social Determinants of Health

Many examples of substantial oral health disparities and inequities are presented in Section 1 of the report. For some conditions and population groups, little improvement has been made, especially among adults and seniors. Section 1 also describes the adverse social, economic, and national security effects of poor oral health, barriers to care, social and commercial determinants of oral health, and related common risk factors. More than the clinical data collected in a typical dental history is needed to understand social determinants and employ local and upstream interventions. The report suggests obtaining social histories from patients to get information about where people live, learn, work, and play. For example, to learn about socioeconomic status, diet, and medications, we want to know not only “What’s in your wallet,” (as touted in a frequent television advertisement) but what’s in your refrigerator? What’s in your medicine cabinet? Telehealth has given clinicians a look inside patients’ homes. Collaboration with social workers, home health aides, and visiting nurses could inform us even more about the home environment. With integrated electronic medical and dental patient records, oral health professionals and medical colleagues can share information. Barriers to integration and assessment of population health outcomes affect many dentists who still use paper records or software specific to dental care that lacks diagnostic codes and interoperability with other health care records systems (6).

The report highlights the need for more information about adolescents and older adults and other understudied population groups. Section 1 describes many diverse, vulnerable populations (eg, people with special health care needs, low health literacy, mental illness, substance abuse disorders; victims of structural racism) who all need to be included in oral health research. Non-English speakers and hard-to-reach populations that have physical and/or financial barriers to traditional dental care are less likely to be recruited and represented in clinical trials, making results less generalizable and interventions less applicable. The applied research agenda being developed by the American Association of Public Health Dentistry (7) and the “Consensus Statement on Future Directions for the Behavioral and Social Sciences in Oral Health,” which is based on an international summit (8), are helpful in setting research and methodologic priorities, including qualitative, implementation, and health systems research.

## Individual and Community Relationships

Knowledge about the interrelationships between oral and systemic health has greatly expanded since the 2000 report. About 60 adverse health conditions have now been shown to be associated with oral health (1), which is part of the rationale for the integration of oral health and primary care. Research will advance our understanding of the mechanisms by which oral and systemic conditions are affected by upstream environmental and social factors, epigenetic factors, and the aging process, both individually and communally. For example, how do external exposures change our microbiomes? Our oral microbiome may be exposed to air containing Sars-CoV-2, water containing protective fluoride, or many kinds of food, beverages, medications, illicit substances, smoked products, and sometimes the biome of close personal contacts. How does the health of a community’s high caries risk groups change with policies such as a tax on sugar-sweetened beverages, Medicaid reimbursement changes, or health promotion efforts to improve oral health literacy and dietary behaviors? To what extent will increased application of value-based health care reimbursement with emphasis on disease prevention, early detection, and minimally invasive care improve oral health? Will the World Health Organization’s addition of dental products (eg, fluoride toothpaste, low-cost silver diamine fluoride, glass ionomer cement) to its Model List of Essential Medicines (9) increase their use to prevent and treat dental caries for under-resourced populations without access to conventional high-cost dental care?

## Scientific Advances and Equitable Distribution

The report’s Section 6 describes many exciting advances in biology, biomimetic dental materials, and technology. Rapid advances in salivary diagnostics are providing information about early, abnormal changes in remote organ systems in the body. Advanced imaging techniques and artificial intelligence can be used for early diagnosis of oral lesions before they are visible to the human eye. The validity and accuracy of these techniques need careful evaluation. Can these earlier clinical end points be used to shorten the length of expensive clinical trials? Guide new preventive strategies? At what point do providers intervene with early preventive or therapeutic strategies instead of letting the body heal itself?

Will populations at greatest risk for disease and the greatest barriers to accessing dental care be able to benefit from early intervention? Every intervention has a cost. If access to new prevention and therapeutic discoveries is not equitable, will health disparities worsen? We need community engagement in the research process and the tools from many disciplines to measure and facilitate the best outcomes. The national Oral Health Progress and Equity Network’s blueprint for improving oral health for all includes 5 levers to advance oral health equity: “amplify consumer voices, advance oral health policy, integrate dental and medical [care], emphasize prevention and bring care to the people” (10).

## Educational Opportunities

Who will analyze all these data mined from many micro and macro sources, and who will interpret the data? Health learning systems and complex software algorithms are being developed to provide automated diagnostic information. Data analysts with knowledge of these and other sophisticated tools and modeling approaches are needed.

The dental, oral, and craniofacial research and practice communities increasingly need to be part of interdisciplinary research and educational programs with opportunities for collaboration and learning. Federally qualified health centers and look-alikes are good sites for medical–dental integration, but many of these facilities do not provide dental care.

More positions are needed for dental public health specialists who can lead advocacy efforts, interdisciplinary teams of researchers, clinicians, and community partners and conduct research. For example, the new Dental Public Health Research Fellowship at the National Institute of Dental and Craniofacial Research will provide more intensive research training to further advance dental public health and population-based research. Mechanisms are needed to promote, facilitate, and reward sharing of research and training resources across disciplines in our competitive environment.

## Summary

Public health perspectives are an important part of interdisciplinary approaches to guide, conduct, and apply research and implement policies to improve oral health. Preventive approaches exist as do barriers to their dissemination and implementation. To prevent disease and improve population oral and overall health, systems change and policy reform are needed along with scientific advances across the research spectrum, more population-level data and analysis, and community participatory engagement. I am optimistic that the next *Oral Health in America* report will describe fewer inequities and more progress toward oral health for all.
